# Diminished ovarian reserve may not be associated with a poorer fresh cycle outcome in women < 38 years

**DOI:** 10.1186/s13048-023-01158-6

**Published:** 2023-04-15

**Authors:** Huizi Jin, Enqi Yan, Dan Chen, Mengya Zhao, Wenju Peng, Yaxin Guo, Lei Jin

**Affiliations:** 1 Reproductive Medicine Center, Tongji Hospital, Tongji Medical College, Huazhong University of Science and Technology, 1095 JieFang Avenue, Wuhan, 430030 People’s Republic of China; 2 Department of Obstetrics and Gynecology, Tongji Hospital, Tongji Medical College, Huazhong University of Science and Technology, Wuhan, China

**Keywords:** Diminished ovarian reserve, IVF/ICSI, Live birth rate, Cumulative live birth rate, Perinatal outcomes

## Abstract

**Background:**

Previous studies have discussed the pregnancy outcomes of diminished ovarian reserve (DOR) patients. However, data on embryonic development potential, neonatal outcomes, and maternal complications of DOR patients still remained unknown. This is the first study to investigate the risk of DOR on pregnancy and perinatal outcomes among women < 38 years.

**Methods:**

Retrospective cohort study was conducted. Patients (< 38 years of age) undergoing their first oocyte retrieval cycle were included. Patients were divided into DOR group and non-DOR group. Pregnancy outcomes of fresh cycle and cumulative live birth rate and perinatal outcomes after one oocyte retrieved cycle were compared between DOR and non-DOR group.

**Result(s):**

From January 2016 to September 2020, there were 8,179 patients involved: 443 patients in the DOR group and 7,736 patients in the non-DOR group. The incidences of live birth and clinical pregnancy did not differ significantly between patients with or without DOR after fresh cycle transfer, but the cumulative live birth rate was significantly lower in DOR group. Among women who had singleton live births, after binary logistic regression, the rates of maternal complications and neonatal outcomes were comparable in the two groups.

**Conclusion(s):**

DOR patients (< 38 years of age) showed similar pregnancy outcomes in the first fresh embryo transfer cycle but a lower chance of live birth after a whole oocyte retrieval cycle to non-DOR patients and DOR is not associated with adverse perinatal outcomes.

**Supplementary Information:**

The online version contains supplementary material available at 10.1186/s13048-023-01158-6.

## Introduction

As women age, their fecundity declines slowly but drops rapidly after the year 37 s [1]. The ability for bearing a child ultimately terminates at menopause on account of follicular pool exhaustion. Diminished ovarian reserve (DOR) is characterized as a loss of fertility prematurely [2]. It is often encountered in clinical practice. By report, the percentage of DOR is 6.3% in patients ≤ 35 years of age [3]. There are a lot of factors that result in DOR, such as ovarian surgery, therapy treatments for cancer, endometriosis, smoking, and infections [4]. With females entering the workforce and pursuing a higher educational degree, all these social and economic issues lead many women to postpone the time of having their first child to an advanced age. DOR surely puts much more burden on childbearing.

The clinical performances of DOR patients have been studied extensively these days. A meta-analysis including 16 studies shows DOR is a risk factor for miscarriage and a meta-analysis reported an apparent association between DOR and recurrent pregnancy loss, meaning DOR may be relevant to a reduction in ovarian quality and oocyte number [5, 6]. Whereas recent studies using preimplantation genetic testing for aneuploidy (PGT-A) argue that DOR patients have equivalent live birth rates to their age-matched controls, implying DOR may not suffer from a decrease in quality [7]. Whether DOR women combined with an additional qualitative penalty is unsettled. Infertile women due to decreased ovarian reserve often turn to fertility treatment and for some patients, the last resort to successfully conceive is using donor oocyte. Hence, the chance of becoming pregnant of DOR patients after ART treatment really deserves studying. In the meantime, despite the abundant literature evaluating pregnancy outcomes, there are a few researches discussing the maternal and neonatal outcomes of DOR patients in fresh cycles to date [3, 8, 9]. Data on embryonic development potential, neonatal outcomes and maternal complications of DOR patients are still limited. The health of perinatal period and neonates also count.

We restricted our subjects to women with age < 38 years before ovarian stimulation according to a previous study [7]. In this study, we aimed to study the pregnancy and perinatal outcomes of DOR patients. We focused on the pregnancy outcomes of fresh cycles of DOR and non-DOR patients who were less than 38 years after the optimal embryo (s) were transferred. Also, we calculated the cumulative live birth rates of each group after one oocyte retrieved cycle. For perinatal outcome analysis, we compared that of single live births which resulted from both fresh and frozen-thawed embryo transfer cycles.

## Materials and methods

### Study population and data collection

We collected the clinical data of patients who underwent their first IVF/ICSI cycle from January 2016 to September 2020 at the Reproductive Medicine Center of Tongji Hospital to conduct this retrospective study. Only data from patients (< 38 years old) without PGD/PGS would be included. Our exclusion criteria were (1) no available embryos were obtained in the current cycle, (2) patients with polycystic ovary syndrome as defined by the 2003 Rotterdam Consensus revised diagnostic criteria [10], (3) uterine cavity abnormalities, (4) chromosomal abnormalities in either of the couple, (5)endometriosis, (6) oocyte totally or partly freezing cycles, (7) patients underwent whole embryos frozen strategies and (8)core data missing (e.g., no information on BMI or endometrial thickness). As a more sensitive marker, AMH has been used most widely to predict reproductive potential. AMH was measured within 12 months before the IVF/ICSI stimulation. The AMH concentration was determined by the electrochemiluminescence immunoassay on day 3 of the menstrual period. After the inclusion and exclusion, which is shown in Supplemental Fig. 1, we divided the patients into DOR group (AMH < 1.1 ng/ML) and non-DOR group (AMH ≥ 1.1 ng/ML) according to the AMH level. For further studies analyzing perinatal outcomes, patients with vanishing twin syndrome or multiple live births were excluded, which means only patients with first singleton live birth (during the study period) were considered, either in the fresh or frozen-thawed embryo transferred (FET) cycles.

This cohort included 8,179 patients. Of them, 443 patients were in the DOR group, and 7,736 were in the non-DOR group. Ultimately, 183 DOR patients and 4,320 non-DOR patients had singleton live births after a whole oocyte retrieved cycle and their perinatal outcomes were further studied.

We obtained the sociodemographic data, baseline information, IVF/ICSI data, and pregnancy outcomes from our electronic medical record system. Perinatal and neonatal information was collected by trained nurses through telephone interviews at specific time points before and after delivery. All information was confirmed by multiple reviews.

### ART treatment

Details on ovarian stimulation, oocyte retrieval, embryo culture, morphological grading, vitrification cryopreservation and warming procedures, and embryo transfer have been described in the previous study [11]. Briefly, oocytes were fertilized by routine IVF or ICSI. We routinely performed ovarian stimulation protocols with gonadotropin-releasing hormone (GnRH) agonist protocols, GnRH antagonist protocols, and other protocols, including mild stimulation protocols and luteal phase stimulation protocols. After observation of follicles larger than 14 mm in diameter, 10,000 IU of HCG was given as a trigger. Oocyte collection would be performed 36–38 h later. After 16–18 h of fertilization, 2PN embryos would continue to be cultured until day 2 or 3, and high-quality cleaved embryos were selected for fresh cycle transfer. Normal fertilization rate was calculated as the number of 2PN embryos/number of mature oocytes. In some cases, embryos continued to be cultured until Day 5, 6, or 7 to obtain blastocysts. Blastocyst formation rate was calculated as the number of blastocysts/number of day 3 embryos for extended culture. Cleavage embryos were evaluated according to 3 parameters: (1) the number of blastomeres, (2) fragmentation rate, and (3) symmetry [12]. Blastocysts were evaluated with reference to the Gardner scoring system [13]. Available blastocysts were defined as those blastocysts at stage 3 and above with a score of B and above of inner cell mass, and the number of them/the total number of blastocysts is the available blastocyst rate. The endometrial preparation protocols utilized in FET cycles were: the natural cycle, the programmed cycle, or the stimulated cycle. In our center, we recommend the programmed cycle for the convenience of scheduling the date of embryo transplantation. The maximum number of embryos to be transferred at one time is two, and both embryos should be from the same stimulation cycle.

### Outcome assessments

Biochemical pregnancy loss was defined as a positive human chorionic gonadotropin (hCG) test at 14 days after embryo transfer without clinical pregnancy on ultrasonography. Clinical pregnancy was determined by the ultrasonographic visualization of one or more gestational sacs. A live birth was defined as the birth of one or more live infant(s) after 24 weeks of gestation.

The main outcome of this study was the live birth rate (LBR). On account of the difference in the embryo type transferred between the DOR and non-DOR groups, we calculated live birth rates and adjusted odds ratios based on the transfer of embryo types, as well as comparing DOR patients with 1 embryo transferred to non-DOR patients. To calculate the cumulative live birth rate for an oocyte retrieval cycle, we followed these patients for two years until all embryos were observed to be used up or live births were obtained, and for those patients who did not reach our defined endpoint after two years, we conservatively estimated that they would not obtain another live birth from the current cycle.

Secondary endpoints were perinatal outcomes and neonatal outcomes. Maternal complications included hypertensive disorders of pregnancy (HDP), gestational diabetes mellitus, and placenta praevia. HDP was diagnosed according to the International Society for the Study of Hypertension in Pregnancy (ISSHP) criteria [14]. HDP includes gestational hypertension, preeclampsia, and eclampsia. Gestational diabetes mellitus was diagnosed according to the International Society for the Study of Diabetes and Pregnancy (ISSHP) [15]. Neonatal outcomes included preterm birth, cesarean section, male gender, low birth weight, macrosomia, and fetal malformation. Preterm birth was defined as a live birth before 37 weeks of gestation and very preterm birth was defined as a live birth before 32 weeks of gestation. Low birth weight was defined as birth weight < 2,500 g for infants delivered at term, and macrosomia was defined as birth weight > 4,000 g for newborns.

### Statistical analysis

Continuous variables were compared using Mann–Whitney U-tests and expressed as medians (first quartile, third quartile). Categorical variables were compared using Chi-square tests or Fisher's exact test when appropriate and were presented as the frequency with proportion.

Because our primary outcome of interest was the LBR, we used two adjusted models to remove the effect of confounding. Binary logistic regression was used in Model 1 and inverse probability weighting (IPW) was used in model 2.

We applied Model 1 and Model 2 with live birth rates as the dependent variable, and outcomes were presented as adjusted odds ratio (aOR) with 95% CIs. Included covariates were female age, BMI, primary infertility, duration of infertility in years, infertility diagnosis, controlled ovarian stimulation (COS) protocols, fertilization methods, number of embryos transferred, type of embryo transferred, and endometrial thickness.

We used binary logistic regression in the analysis of singleton abnormal perinatal outcomes. Included covariates were female age, BMI, primary infertility, duration of infertility in years, infertility diagnosis, COS protocols, fertilization methods, number of embryos transferred, type of embryo transferred, and from either fresh or FET cycles.

All of these calculations were analyzed by SPSS 26.0 (IBM, Chicago, IL) and the statistical packages R (v.4.1; R Foundation for Statistical Computing, Vienna, Austria). *P* value < 0.05 was considered statistically significant.

## Results

### Participants’ characteristics and fertility evaluations

**Fig. 1:**
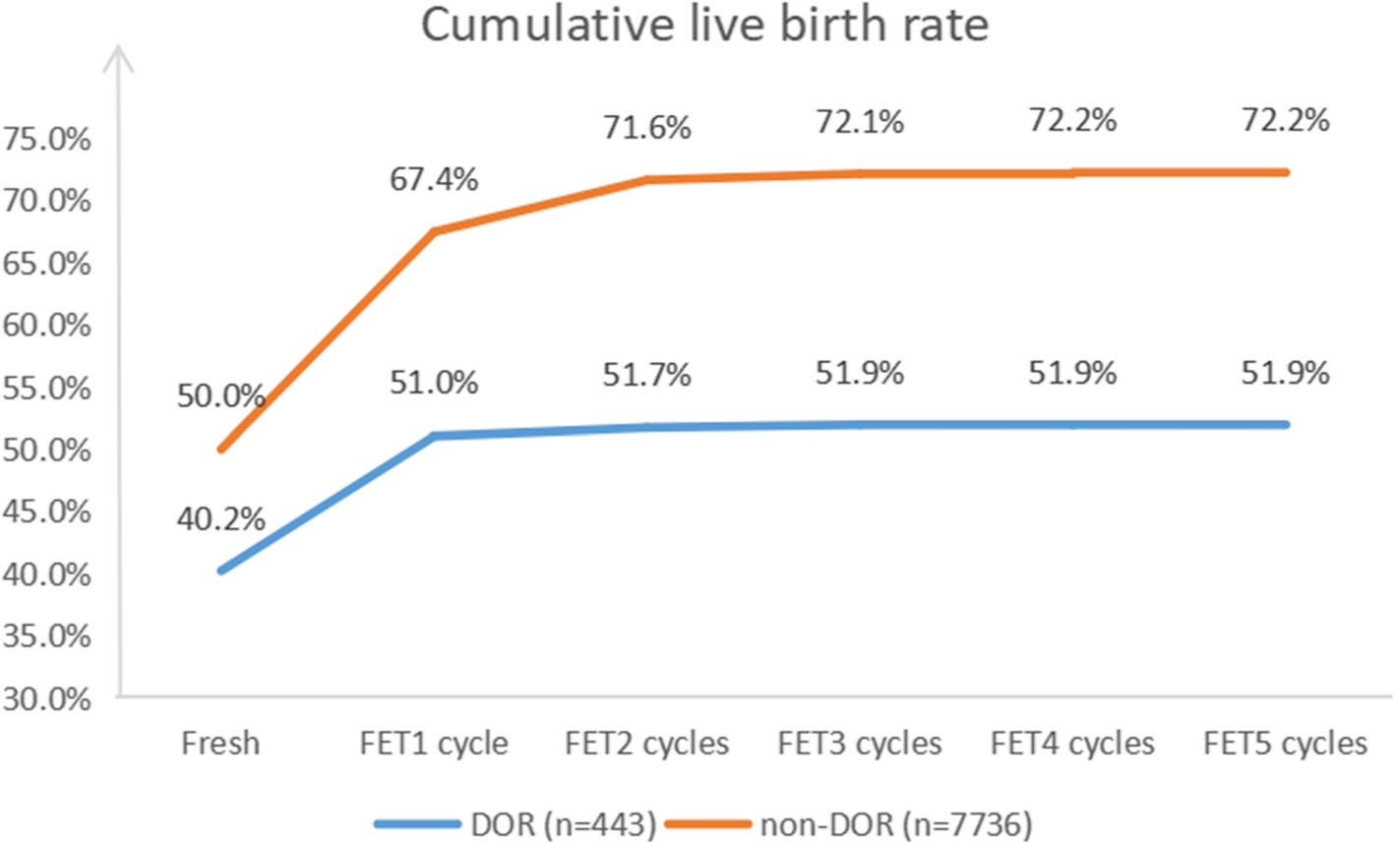
The cumulative live birth rates of the two groups

After screening, 8,179 cycles from January 2016 to September 2020 in Tongji hospital were included. According to the AMH level, these patients were subsequently classified into the DOR group (AMH < 1.1 ng/mL, *n* = 443) and the non-DOR group (AMH ≥ 1.1 ng/mL, *n* = 7,736) (Fig. 1).

The baseline characteristics and treatments are displayed in Table 1. Overall, DOR patients were older (*P* < 0.001) and heavier (*P* = 0.048) compared to controls. However, the infertility duration of DOR patients was shorter (*P* = 0.004). As presumed, the basal AMH and AFC were significantly lower in DOR patients (*P* < 0.001). GnRH antagonist protocol was usually used in the DOR group (83.1% [368/443]), while GnRH agonist protocol was mostly used in the non-DOR group (76.6% [5,928/7,736]). Patients in the DOR group experienced shorter Gn stimulation and received higher Gn doses and still obtained significantly fewer oocytes than the control group. Female factors were more common in DOR patients (67.7% [300/443] vs. 49.3% [3,816/7,736]) while there was a significantly higher rate of male infertility in non-DOR patients (5.4% [24/443] vs. 24.1% [1,868/7,736]), and this led to the differences in the fertilization methods (*P* = 0.001).

**Table 1:** Patients’ general characteristics and clinical outcomes

	**DOR (*n* = 443)**	**Non-DOR (*n* = 7736)**	***P* value**
**Baseline characteristics**			
Age (y)	31(28–34)	30(27–32)	< 0.001*
BMI (kg/m^2^)	21.6(19.8–24.0)	21.3(19.6–23.4)	0.048*
Primary infertility	303(68.4%)	5546(71.7%)	0.135
Duration of infertility (years)	2(1–4)	3(2–4)	0.004*
AMH (ng/mL)	0.8(0.6–1.0)	4.1(2.6–6.3)	< 0.001*
AFC	6(4–8)	13(9–17)	< 0.001*
Infertility diagnosis			< 0.001*
Female factor	300(67.7%)	3816(49.3%)	
Male factor	24(5.4%)	1868(24.1%)	
Female and male factors	111(25.1%)	1164(15.0%)	
Unknown	8(1.8%)	888(11.5%)	
Prior ovarian surgery (n)	7(1.6%)	96(1.2%)	0.534
COS Protocol			< 0.001*
GnRH agonist	60(13.5%)	5928(76.6%)	
GnRH antagonist	368(83.1%)	1758(22.7%)	
Others	15(3.4%)	50(0.6%)	
Gn vials (days)	9(8–10)	10(9–11)	< 0.001*
Total Gn dose (IU)	2775(2400–3422)	2250(1800–2873)	< 0.001*
E2	1356 (964–1933)	2437 (1666–3417)	< 0.001*
P	0.64 (0.44–0.91)	0.81 (0.58–1.05)	< 0.001*
Endometrial thickness	10.9 (9.4–12.4)	11.8 (10.3–13.5)	< 0.001*
Fertilization methods			0.001*
IVF cycles	324(73.1%)	5021(64.9%)	
ICSI cycles	93(21.0%)	2235(28.9%)	
IVF + ICSI cycles	26(5.9%)	480(6.2%)	
No. of oocytes retrieved	6(4–8)	12(9–15)	< 0.001*
Mature oocyte rate (%)	2335/2652(88.0%)	80,849/93173(86.8%)	0.056
Normal fertilization rate (%)	1702/2335(72.9%)	56,529/80849(69.9%)	0.002*
Blastocyst formation rate (%)	622/986(63.1%)	29,790/44602(66.8%)	0.015*
Available blastocyst rate (%)	418/622(67.2%)	19,574/29790(65.7%)	0.437
Embryo type transferred			< 0.001*
Cleavage embryo	425 (95.9%)	7051 (91.1%)	
Blastocyst	18 (4.1%)	685 (8.9%)	
2 embryos transferred	162 (36.6%)	3388 (43.8%)	0.003*
No. of embryo transferred	605	11,124	
Biochemical pregnancy rate (%)	20 (4.5%)	393 (5.1%)	0.597
Clinical pregnancy rate (%)	212 (47.9%)	4440 (57.4%)	< 0.001*
Implantation rate of cleavage embryos (%)	242/585 (41.4%)	4974/10407 (47.8%)	0.002*
Implantation rate of blastocysts (%)	10/20 (50.0%)	423/717 (59.0%)	0.420
Live birth rate (%)	178 (40.2%)	3866 (50.0%)	< 0.001*
Cumulative live birth rate (%)	230 (51.9%)	5589 (72.2%)	< 0.001*

Continuous data are reported as medians (first quartile, third quartile) and analyzed by Mann–Whitney U tests

Categorical data are reported as n (%) and analyzed by χ^2^

*DOR* diminished ovarian reserve, *BMI* body mass index, *AMH* anti-Müllerian hormone, *AFC* antral follicle count, *COS* controlled ovarian stimulation, *Gn* gonadotropin, *GnRH* gonadotropin-releasing hormone, *E2* estradiol, *P* progesterone, *IVF* in vitro fertilization, *ICSI* intracytoplasmic sperm injection

^*^P < .05

Besides that, comparisons between the two populations showed no significant differences concerning primary infertility and prior ovarian surgeries.

### Laboratory outcomes

Laboratory outcomes were shown in Table 1. The number of oocytes obtained was significantly lower in patients with DOR than in controls (P < 0.001). But the mature oocyte rate was similar between groups. Additionally, the normal fertilization ability of mature oocytes (normal fertilization rate) was even higher in the DOR group (72.9% vs. 69.9%, *P* = 0.002) while they had a lower blastocyst formation rate (63.1% vs. 66.8%, *P* = 0.015), despite a comparable available blastocyst rate.

### Pregnancy outcomes

Tables 1 and 2 show the pregnancy outcomes of the two groups. The frequency of transferring blastocysts was much higher in women without DOR (8.9% [685/7,736] vs. 4.1% [18/443]; *P* < 0.001). Fewer double embryo transfers were performed in the DOR group (36.6% [162/443] vs. 43.8% [3,388/7,736]). The implantation rate of cleavage embryos remains lower in DOR group (41.4% [242/585] vs. 47.8% [4,974/10,407]; *P* = 0.002) while the implantation rate of blastocysts differed no significantly. Apart from that, the DOR group had a lower clinical pregnancy rate (CPR) and LBR (47.9% [212/443] vs. 57.4% [4,440/7,736]; *P* < 0.001 and 40.2% [178/443] vs. 50.0% [3,866/7,736]; *P* < 0.001).

**Table 2:** Odds ratios and adjusted odds ratios of pregnancy outcomes before and after binary logistic regression and IPW

	**Live birth**	**Crude Model**^**a**^		**Adjusted Model 1**^**b**^		**Adjusted Model 2**^**c**^	
	**n (%)**	**OR (95%CI)**	***P* value**	**Adjusted OR (95%CI)**	***P*(a) value1**	**Adjusted OR (95%CI)**	***P*(a) value2**
Fresh cycles^d^							
Groups							
DOR (*n* = 443)	178(40.2%)	0.672(0.553–0.816)	< 0.001*	0.969 (0.785–1.195)	0.766	0.934 (0.750–1.164)	0.545
Non-DOR (*n* = 7,736)	3866(50.0%)	Reference		Reference		Reference	
Blastocyst transferred^e^							
DOR (*n* = 18)	8 (44.4%)	0.697 (0.272–1.788)	0.453	1.454 (0.509–4.152)	0.485	0.504 (0.097–2.057)	0.359
Non-DOR (*n* = 685)	366 (53.4%)	Reference		Reference		Reference	
Cleavage embryo transferred^e^							
DOR (*n* = 425)	170 (40.0%)	0.676 (0.553–0.825)	< 0.001*	0.956 (0.772–1.185)	0.956	0.931 (0.745–1.162)	0.527
Non-DOR (*n* = 7,051)	3502 (49.7%)	Reference		Reference		Reference	
One embryo transferred^f^							
DOR with one embryo transferred (*n* = 281)	105 (37.4%)	0.694 (0.541–0.890)	0.004*	1.001 (0.765–1.310)	0.994	0.727 (0.550–0.956)	0.024*
Non-DOR with one embryo transferred (*n* = 4348)	2010 (46.2%)	Reference		Reference		Reference	
Two embryos transferred^f^							
DOR with two embryos transferred (*n* = 162)	73 (45.1%)	0.675 (0.492–0.927)	0.015*	0.907 (0.648–1.270)	0.571	1.228 (0.860–1.764)	0.220
Non-DOR with two embryos transferred (*n* = 3388)	1856 (54.8%)	Reference		Reference		Reference	
Cumulative live birth^g^							
DOR (*n* = 443)	230 (51.9%)	0.414 (0.342–0.502)	< 0.001*	0.604(0.491–0.744)	< 0.001*	0.635 (0.510–0.794)	< 0.001*
Non-DOR (*n* = 7,736)	5589 (72.2%)	Reference		Reference		Reference	

^a^Odds ratios (ORs), 95% confidence intervals (CIs), and *P* values were based on the univariate analysis

^b^ Adjusted odds ratios (aORs), 95% confidence intervals (CIs), and *P*(a) values1 were based on the binary logistic regression

^c^ Adjusted odds ratios (aORs), 95% confidence intervals (CIs), and *P*(a) values2 were based on the inverse probability weighting

^d^ adjusted for female age, BMI, primary infertility, duration of infertility in years, infertility diagnosis, COS protocols, fertilization methods, number of embryos transferred, type of embryo transferred, and endometrial thickness

^e^ adjusted for female age, BMI, primary infertility, duration of infertility in years, infertility diagnosis, COS protocols, fertilization methods, number of embryos transferred, and endometrial thickness

^f^ adjusted for female age, BMI, primary infertility, duration of infertility in years, infertility diagnosis, COS protocols, fertilization methods, number of embryos transferred, type of embryo transferred, and endometrial thickness

^g^ adjusted for female age, BMI, primary infertility, duration of infertility in years, infertility diagnosis, COS protocols, and fertilization methods

*DOR* diminished ovarian reserve

^*^P < .05

After adjusting the likely impact of covariates in adjusted Model 1 (binary logistic regression) and adjusted Model 2 (inverse probability weighting), there were no significant differences observed between the two populations in regard to live birth. We performed a subgroup analysis according to the type of embryos transferred; the results remained consistent. Also, the LBR of two-embryo-transferred patients in the two groups differed not significantly. However, among patients who had one embryo transferred, the two adjustment models suggested opposite conclusions——Model 1 led to no significant difference between DOR and Non-DOR patients while Model 2 suggested a significant decrease in LBR in DOR patients (Table 2).

For secondary outcomes, adjusted model 1 and adjusted model 2 were used to calculate adjusted ratio ratios for the occurrence of biochemical pregnancy, clinical pregnancy, ectopic pregnancy, and pregnancy loss events in fresh cycles in patients with DOR, as shown in Supplemental Table 1. The probability of clinical pregnancy, ectopic pregnancy, and miscarriage in patients with DOR was not significantly different from that in patients with Non-DOR, and model 2 suggested that DOR was a risk factor for the occurrence of biochemical pregnancy while Model 1 led to no significant difference.

As for cumulative outcomes after one entire ART cycle, the cumulative live birth rate of DOR group was 51.9%, while that of non-DOR group was 72.2% (*P* < 0.001). The difference between the two groups was statistically significant both before and after adjustment. Specific information on the fresh and FET cycles is shown in Supplemental Table 2 and Fig. 1.

### Singleton perinatal outcomes

As shown in Table 3, after excluding women with stillbirths, women with vanishing twin syndrome, or live births from multiple pregnancies, there were 183 women (141 from fresh cycles and 42 from FET cycles) with DOR and 4,320 women without DOR who had singleton live births (2,949 from fresh cycles and 1,371 from FET cycles). There were significant differences in terms of age, BMI, male factor infertility, and type of embryo transferred between DOR patients and non-DOR patients. There was no significant difference in transfer of 2 embryos between the two groups.

**Table 3:** Singleton abnormal perinatal outcome

	**Case (*n* = 183)**	**Control (*n* = 4320)**	***P* value**	***P*(a) value**
Fresh cycles	141 (77.0%)	2949 (68.3%)	0.012*	-
FET cycles	42 (23.0%)	1371 (31.7%)		-
Age	31 (28–33)	30 (27–32)	< 0.001*	-
BMI	21.9 (19.9–24.1)	21.3 (19.6–23.4)	0.034*	-
Male factor infertility	14 (7.7%)	1056 (24.4%)	< 0.001*	-
Two embryos transferred	48 (26.2%)	1361 (31.5%)	0.132	-
Blastocyst	8 (4.4%)	485 (11.2%)	0.004*	-
HDP	9 (4.9%)	121 (2.8%)	0.094	0.202
Gestational diabetes mellitus	14 (7.7%)	250 (5.8%)	0.293	0.521
Abnormal placenta	4 (2.2%)	165 (3.8%)	0.255	0.277
Cesarean delivery	138 (75.4%)	3206 (74.2%)	0.717	0.684
Preterm delivery, < 37 wk	11 (6.0%)	261 (6.0%)	0.986	0.872
Very preterm delivery, < 32wk	1 (0.5%)	25 (0.6%)	0.955	0.869
Male gender	89 (48.6%)	2357 (54.6%)	0.115	0.096
Low birth weight, < 2,500 g	1 (0.5%)	49 (1.1%)	0.457	0.512
Macrosomia, > 4000 g	14 (7.7%)	204 (4.7%)	0.071	0.140
Fetal malformation	3 (1.6%)	47 (1.1%)	0.486	0.940

Continuous data are reported as medians (first quartile, third quartile) and analyzed by Mann–Whitney U tests

Categorical data are reported as n (%) and analyzed by χ^2^

*P*(a) values are based on binary logistic regression, adjusted for female age, BMI, primary infertility, duration of infertility in years, infertility diagnosis, COS protocols, fertilization methods, number of embryos transferred, type of embryo transferred, and from either fresh or FET cycles

*HDP* hypertensive disorders of pregnancy

^*^P < .05

As for perinatal complications, there were no significant differences between the two groups in gestational diabetes mellitus and gestational hypertension and placental abnormalities. No significant differences were found in cesarean delivery or gender of newborns between DOR and non-DOR. Also, neonatal complications consisting of preterm birth, low birth weight, and macrosomia or fetal malformation were comparable in DOR patients and non-DOR patients. There was no difference in neonatal complications between the two groups.

Singleton live birth outcomes for fresh cycles alone are shown in Supplemental Table 3. After using a binary logistic regression model, the difference in perinatal complications between the two groups was not statistically significant.

## Discussion

In the present study, we investigated the risk of DOR on pregnancy and perinatal outcomes among women < 38 years. According to our results, DOR patients had a similar LBR after fresh ET cycles but a significantly lower CLBR after a whole oocyte retrieved cycles than non-DOR patients. Further, DOR did not increase the risk of perinatal complications in comparison to non-DOR.

Multiple clinical and experimental researches have proved that women’s ovaries would experience a physiologically sharp decline in both the quantitative and qualitative aspects around the age of 38. The aneuploidy rate of embryos substantially rises when reaching 38 years of age [16], and at the same time, data obtained from the mathematical model displayed the primordial follicles would fall below the threshold at approximately 37.5 years among most people [17, 18]. Therefore, in the current study, we restricted our study subjects to women less than 38 years in order to figure out whether DOR patients suffer from a simultaneous qualitative reduction besides a decreased oocyte number.

Our results showed DOR patients have a similar clinical pregnancy rate and LBR in the fresh cycle. This can somewhat indicate that DOR women are not associated with a decreased oocyte quality. The average of oocytes acquired was significantly lower in DOR patients. However, we found that the oocyte maturation rate and fertilization rate were not decreased in DOR women. Attrition occurs at each stage in the IVF process. Therefore, non-DOR patients could have much more chance of acquiring considerable embryos. Non-DOR patients tend to transplant more than one embryo to obtain much more opportunities of getting pregnant. It is also worth noting that the proportion of blastocyst transfer in non-DOR patients was significantly higher. The few number of oocyte limited the blastocyst transplantation to some degree. Compared to cleavage embryo, blastocyst transfer is considered to be more physiological because it is closer to natural conception. Transfer at the blastocyst stage can improve the synchronicity of both uterus and embryo and therefore lead to higher LBRs [19]. Therefore, we conducted subgroup analyses among women who had one embryo transferred and women who had blastocyst transferred.

In agreement with us, Morin et al. [7] found the LBR of patients with AMH in the < 10th percentile and patients with AMH in the 25th to 75th percentile was compared among all patients and among patients who used PGT-A after blastocyst transfer. In a recent study performed by Fouks et al. [20], young DOR patients were not associated with a reduced euploid rate compared to their age-matched counterparts and the LBR was not significantly different between the two groups after transferring euploid single-embryo. However, there are some publications that provided the opposite views. Jaswa et al. [21] included more than 1,000 patients aged 19–42 years and found the euploid rate of DOR patients was lower than non-DOR patients at different maternal age subgroups. What’s more, Shahine et al. [22] found the aneuploidy rate was higher in DOR patients who complained about recurrent pregnancy loss. The application of PGT-A is under a strict condition and it is not universally used in all infertile patients, therefore, this result is not representative of the broader picture. The study by Tiegs et al. [23] showed similar pregnancy outcomes between young patients with AMH < 1.0 ng/mL and AMH ≥ 1.0 ng/mL. Though it was performed in women using intrauterine insemination (IUI) rather than IVF, it also suggested the quantitative, but not qualitative distinction of DOR versus non-DOR, similar to our findings. However, there are data showing different opinions. Chang et al. [24] included 305 young women with DOR, 279 aged women with DOR, and 821 young women with a normal ovarian reserve who had embryos transferred. They found a markedly lower rate of biochemical pregnancy and clinical pregnancy of young DOR women in comparison to women with normal ovarian reserve. However, they did not adjust for clinical outcomes by type and number of embryos transferred, whereas our study did, which made more credibility. Zakhari et al. [25] performed a single ideal blastocyst transfer in women < 40 years old. They defined DOR as AFC ≤ 5 follicles. The biochemical pregnancy rate, CPR, and LBR were superior in women with AFC > 5 follicles. Earlier publications indicate a likely association between DOR and recurrent pregnancy loss [5, 22, 26]. Existing evidence is contradicting and far from conclusive. Our results imply that the developmental potential of embryo in young DOR patients may be as good as in non-DOR patients.

In the present study, we found DOR did not increase the risk of perinatal complications. Published data didn’t show a connection between DOR and abnormal neonatal outcomes. However, there are studies indicating the association between DOR and a higher risk of HDP. Han et al.[8] found that the rate of HDP was significantly higher in DOR women under 40 years old compared to their age-matched controls when fresh ET was performed. They held that ovarian aging is connected with abnormal luteal phase function and this may lead to vascular problems and hypertensive disorders during pregnancy [27–29]. A newly published data conducted by Herman et al. [9] found a higher risk of preeclampsia during pregnancy in DOR patients. The possible reason is that low AMH value is a phenotype of insulin resistance which is related to vascular damage, thus leading to an increased likelihood of HDP in DOR patients [30]. Further, low AMH may lead to hormonal disorders and an increased rate of obesity, therefore increasing the risk of cardiovascular disease [31, 32]. While we didn’t find significant differences in the incidence of HDP whether in fresh cycles or in a whole oocyte retrieval cycle. We assumed this may be caused by the difference in the study populations because patients in our cohort were apparently younger than that in those studies because age is the dependent factor of abnormal perinatal outcomes. In fact, the rate of HDP of DOR patients under 35 years was similar to that of normal ovarian reserve patients in Han’s work. What’s more, whether DOR is associated with luteal dysfunction or metabolic disorder is far from conclusive [33]. In our study, a part of the singleton live births was from FET cycles. The programmed FET cycles accounted for more than 90% of endometrial preparation protocols used in both the DOR and non-DOR groups, which was reported to have a higher risk of HDP, postpartum hemorrhage, cesarean section, and giving birth of a newborn being large for gestational age compared to natural cycle [34, 35]. However, the programmed FET was used in a similar proportion between the two groups and we adjusted the endometrial preparation protocol as a confounding factor. Well-organized prospective research is needed to investigate the possible association between DOR patients and cardiovascular risk factors and if the notable link is established, this can contribute to enhancing patient care post-conception.We focused on fresh cycles and aimed to evaluate the LBR to investigate whether the embryo quality is decreased in the DOR group. When embryo transfers take place, the optimal embryo was the first priority. We also included all of the cycles with embryo transferred after a whole oocyte retrieval cycle. Thus, one of the strengths of this study is that we interpreted the qualitative and quantitative performances at the same time. Another strength of this study is that we adopted multiple statistical methods to make our results more reliable. However, there are several limitations. Firstly, the number of blastocyst embryos transferred was relatively fewer. We would collect this part of data prospectively. We found no statistically significant difference in LBR when transferring blastocyst which we assumed was caused by the limited number of patients who had blastocyst transfer. Then, we only included patients who had available embryos and this may overestimate the fertility fecundity of DOR patients. Finally, due to its retrospective nature, our study was unable to provide information on maternal smoking or the weight gained during gestation.

Young DOR women are not always linked with a poor response [36]. The number of oocytes acquired is not unacceptable for some patients. However, according to our results, we provided a signal to them, age should also be taken into consideration for predicting fecundity primarily. The diagnosis of DOR questions the probability of bearing a child. In the light of the present study, DOR may not be connected to both poorer oocyte quality. Young DOR women with certain causes of infertility should seek medical advice immediately and the pregnancy and perinatal outcomes are somewhat encouraging.

## Supplementary Information

**Additional file 1: Supplemental Figure 1.** Flowchart of participants.

**Additional file 2: Supplemental Table 1.** Odds ratios and adjusted odds ratios of pregnancy outcomes before and after binary logistic regression and IPW.

**Additional file 3: Supplemental Table 2.** Cumulative outcomes after one entire ART cycle including fresh and all subsequent frozen embryo transfer cycles in the two groups.

**Additional file 4: Supplemental Table 3.** Singleton abnormal perinatal outcome in fresh cycles.

## Data Availability

The data underlying this article will be shared on reasonable request to the corresponding author.

## References

[1] American College of Obstetricians and Gynecologists Committee on Gynecologic Practice and Practice Committee (2014). Female age-related fertility decline. Committee Opinion No. 589. Fertil Steril Am Soc Reprod Med.

[2] Pastore LM, Christianson MS, Stelling J, Kearns WG, Segars JH (2018). Reproductive ovarian testing and the alphabet soup of diagnoses: DOR, POI, POF, POR, and FOR. J Assist Reprod Genet J Assist Reprod Gen.

[3] Hu S, Xu B, Jin L (2020). Perinatal outcome in young patients with diminished ovarian reserve undergoing assisted reproductive technology. Fertil Steril Elsevier Inc.

[4] Peuranpää P, Hautamäki H, Halttunen-Nieminen M, Hydén-Granskog C, Tiitinen A (2020). Low anti-Müllerian hormone level is not a risk factor for early pregnancy loss in IVF/ICSI treatment. Hum Reprod.

[5] Bunnewell SJ, Honess ER, Karia AM, Keay SD, Al Wattar BH, Quenby S (2020). Diminished ovarian reserve in recurrent pregnancy loss: a systematic review and meta-analysis. Fertil Steril Elsevier Inc.

[6] Busnelli A, Somigliana E, Cirillo F, Levi-Setti PE (2021). Is diminished ovarian reserve a risk factor for miscarriage? Results of a systematic review and meta-analysis. Hum Reprod Update.

[7] Morin SJ, Patounakis G, Juneau CR, Neal SA, Scott RT, Seli E (2018). Diminished ovarian reserve and poor response to stimulation in patients <38 years old: a quantitative but not qualitative reduction in performance. Hum Reprod.

[8] Han S, Zhai Y, Guo Q, Qin Y, Liu P (2021). Maternal and neonatal complications in patients with diminished ovarian reserve in in-vitro fertilization/intracytoplasmic sperm injection cycles. Front Endocrinol (Lausanne).

[9] Herman HG, Volodarsky-Perel A, Ton Nu TN, Machado-Gedeon A, Cui Y, Shaul J, et al. Diminished ovarian reserve is a risk factor for preeclampsia and placental malperfusion lesions. Fertil Steril. Am Soc Reprod Med 2023 10.1016/j.fertnstert.2023.01.029 10.1016/j.fertnstert.2023.01.029 36702344

[10] Rotterdam ESHRE/ASRM-Sponsored PCOS Consensus Workshop Group (2004). Revised 2003 consensus on diagnostic criteria and long-term health risks related to polycystic ovary syndrome. Fertil Steril.

[11] Wang M, Xi Q, Yang Q, Li Z, Yang L, Zhu L (2021). The relationship between a novel evaluation parameter of premature luteinization and IVF outcomes. Reprod Biomed Online Elsevier Ltd.

[12] Braga DPAF, Setti AS, Figueira RCS, Iaconelli A, Borges E (2014). The importance of the cleavage stage morphology evaluation for blastocyst transfer in patients with good prognosis. J Assist Reprod Genet.

[13] Gardner DK, Schoolcraft WB (1999). Culture and transfer of human blastocysts. Curr Opin Obstet Gynecol.

[14] Tranquilli AL, Dekker G, Magee L, Roberts J, Sibai BM, Steyn W (2014). The classification, diagnosis and management of the hypertensive disorders of pregnancy: a revised statement from the ISSHP. Pregnancy Hypertens An Int J Women’s Cardiovasc Heal.

[15] Metzger BE, Gabbe SG, Persson B, Buchanan TA, Catalano PA, International Association of Diabetes and Pregnancy Study Groups Consensus Panel (2010). International association of diabetes and pregnancy study groups recommendations on the diagnosis and classification of hyperglycemia in pregnancy. Diabetes Care.

[16] Franasiak JM, Forman EJ, Hong KH, Werner MD, Upham KM, Treff NR (2014). The nature of aneuploidy with increasing age of the female partner: A review of 15,169 consecutive trophectoderm biopsies evaluated with comprehensive chromosomal screening. Fertil Steril Elsevier Inc.

[17] Faddy MJ, Gosden RG (1995). Physiology: a mathematical model of follicle dynamics in the human ovary. Hum Reprod.

[18] Gougeon A, Ecochard R, Thalabard JC (1994). Age-related changes of the population of human ovarian follicles: Increase in the disappearance rate of non-growing and early-growing follicles in aging women. Biol Reprod.

[19] Glujovsky D, QuinteiroRetamar AM, Alvarez Sedo CR, Ciapponi A, Cornelisse S, Blake D (2022). Cleavage-stage versus blastocyst-stage embryo transfer in assisted reproductive technology. Cochrane Database Syst Rev.

[20] Fouks Y, Penzias A, Neuhausser W, Vaughan D, Sakkas D (2022). A diagnosis of diminished ovarian reserve does not impact embryo aneuploidy or live birth rates compared to patients with normal ovarian reserve. Fertil Steril Am Soc Reprod Med.

[21] Jaswa EG, McCulloch CE, Simbulan R, Cedars MI, Rosen MP (2021). Diminished ovarian reserve is associated with reduced euploid rates via preimplantation genetic testing for aneuploidy independently from age: evidence for concomitant reduction in oocyte quality with quantity. Fertil Steril Elsevier Inc.

[22] Shahine LK, Marshall L, Lamb JD, Hickok LR (2016). Higher rates of aneuploidy in blastocysts and higher risk of no embryo transfer in recurrent pregnancy loss patients with diminished ovarian reserve undergoing in vitro fertilization. Fertil Steril Elsevier Inc.

[23] Tiegs AW, Sun L, Scott RT, Goodman LR (2020). Comparison of pregnancy outcomes following intrauterine insemination in young women with decreased versus normal ovarian reserve. Fertil Steril Elsevier Inc.

[24] Chang Y, Li J, Li X, Liu H, Liang X (2018). Egg quality and pregnancy outcome in young infertile women with diminished ovarian reserve. Med Sci Monit.

[25] Zakhari A, Ates S, Shaulov T, Dahan MH (2018). Does ovarian reserve affect outcomes in single ideal blastocyst transfers in women less than 40 years of age? Arch Gynecol Obstet. Springer, Berlin Heidelberg.

[26] Trout SW, Seifer DB (2000). Do women with unexplained recurrent pregnancy loss have higher day 3 serum FSH and estradiol values?. Fertil Steril.

[27] von Versen-Höynck F, Schaub AM, Chi Y-Y, Chiu K-H, Liu J, Lingis M (1979). Increased preeclampsia risk and reduced aortic compliance with in vitro fertilization cycles in the absence of a corpus luteum. Hypertens (Dallas, Tex 2019).

[28] von Versen-Höynck F, Narasimhan P, Selamet Tierney ES, Martinez N, Conrad KP, Baker VL (1979). Absent or excessive corpus luteum number is associated with altered maternal vascular health in early pregnancy. Hypertens (Dallas, Tex 2019).

[29] Practice Committees of the American Society for Reproductive Medicine and the Society for Reproductive Endocrinology and Infertility (2021). Diagnosis and treatment of luteal phase deficiency: a committee opinion. Fertil Steril Am Soc Reprod Med.

[30] Park HT, Cho GJ, Ahn KH, Shin JH, Kim YT, Hur JY (2010). Association of insulin resistance with anti-Mullerian hormone levels in women without polycystic ovary syndrome (PCOS). Clin Endocrinol (Oxf).

[31] Tehrani FR, Erfani H, Cheraghi L, Tohidi M, Azizi F (2014). Lipid profiles and ovarian reserve status: a longitudinal study. Hum Reprod.

[32] Woldringh GH, Frunt MHA, Kremer JAM, Spaanderman MEA (2006). Decreased ovarian reserve relates to pre-eclampsia in IVF/ICSI pregnancies. Hum Reprod.

[33] Pfister A, Crawford NM, Steiner AZ (2019). Association between diminished ovarian reserve and luteal phase deficiency. Fertil Steril.

[34] Asserhøj LL, Spangmose AL, AarisHenningsen AK, Clausen TD, Ziebe S, Jensen RB (2021). Adverse obstetric and perinatal outcomes in 1,136 singleton pregnancies conceived after programmed frozen embryo transfer (FET) compared with natural cycle FET. Fertil Steril Elsevier Inc.

[35] Wang B, Zhang J, Zhu Q, Yang X, Wang Y (2020). Effects of different cycle regimens for frozen embryo transfer on perinatal outcomes of singletons. Hum Reprod.

[36] Devine K, Mumford SL, Wu M, DeCherney AH, Hill MJ, Propst A (2015). Diminished ovarian reserve in the United States assisted reproductive technology population: diagnostic trends among 181,536 cycles from the society for assisted reproductive technology clinic outcomes reporting system. Fertil Steril Elsevier Inc.

