# Supplementary figures and images for: Diminished ovarian reserve may not be associated with a poorer fresh cycle outcome in women < 38 years
Source: J Ovarian Res. 2023 Apr 15;16:77. doi: 10.1186/s13048-023-01158-6 (PMC10105451; doi:10.1186/s13048-023-01158-6)

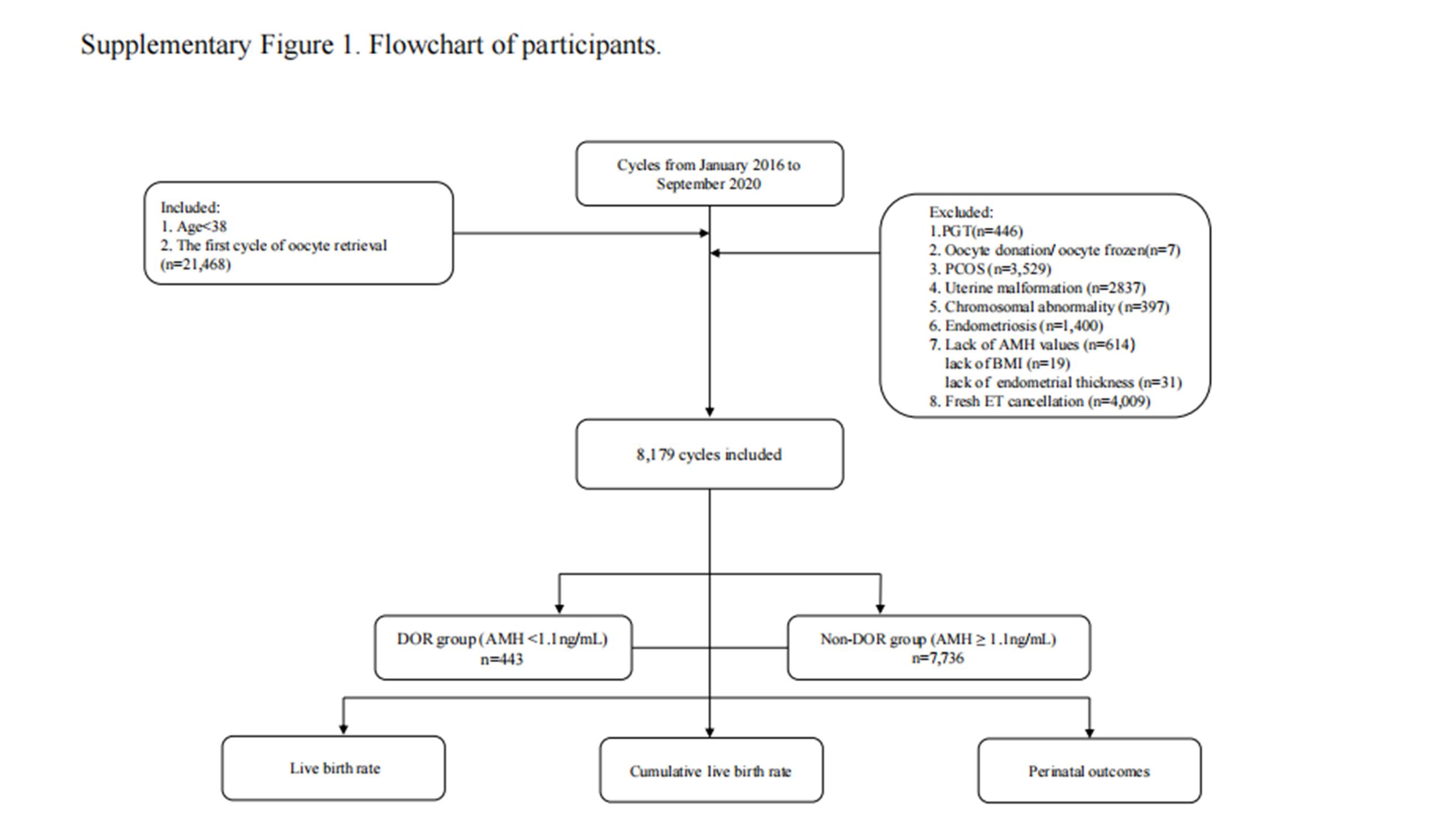

Supplement: Supplementary file 1 — Additional file 1: Supplemental Figure 1. Flowchart of participants. [file 13048_2023_1158_MOESM1_ESM.tif]
