# Supplementary material for: Diminished ovarian reserve may not be associated with a poorer fresh cycle outcome in women < 38 years
Source: J Ovarian Res. 2023 Apr 15;16:77. doi: 10.1186/s13048-023-01158-6 (PMC10105451; doi:10.1186/s13048-023-01158-6)
Supplement: Supplementary file 2 — Additional file 2: Supplemental Table 1. Odds ratios and adjusted odds ratios of pregnancy outcomes before and after binary logistic regression and IPW. [file 13048_2023_1158_MOESM2_ESM.docx]

**Supplemental Table 1. Odds ratios and adjusted odds ratios of pregnancy outcomes before and after binary logistic regression and IPW.**

|  | **Occurrence frequency** | | **Crude Model^a^** | | **Adjusted Model 1^b^** | | **Adjusted Model 2^c^** | |
| --- | --- | --- | --- | --- | --- | --- | --- | --- |
|  | **DOR**  **443** | **Non-DOR**  **7736** | **OR (95%CI)** | **P value** | **Adjusted OR (95%CI)** | **P(a) value1** | **Adjusted OR (95%CI)** | **P(a) value2** |
| Biochemical pregnancy (%) | 20 (4.5%) | 393 (5.1%) | 0.883 (0.540~1.362) | 0.597 | 0.861 (0.529~1.401) | 0.547 | 2.366 (1.687~3.246) | **<0.001** |
| Clinical pregnancy (%) | 212 (47.9%) | 4440 (57.4%) | 0.681 (0.562~0.825) | **<0.001** | 0.959 (0.781~1.179) | 0.694 | 0.902 (0.736~1.108) | 0.325 |
| Ectopic pregnancy (%) | 3 (0.7%) | 69 (0.9%) | 0.758 (0.185~2.044) | 0.639 | 0.433 (0.131~1.433) | 0.170 | 0.937 (0.249~2.438) | 0.908 |
| Pregnancy loss (%) | 34 (16.0%) | 574 (12.9%) | 1.292 (0.872~1.859) | 0.184 | 0.955 (0.638~1.429) | 0.822 | 0.935 (0.584~1.432) | 0.768 |
