# Supplementary material for: Diminished ovarian reserve may not be associated with a poorer fresh cycle outcome in women < 38 years
Source: J Ovarian Res. 2023 Apr 15;16:77. doi: 10.1186/s13048-023-01158-6 (PMC10105451; doi:10.1186/s13048-023-01158-6)
Supplement: Supplementary file 3 — Additional file 3: Supplemental Table 2. Cumulative outcomes after one entire ART cycle including fresh and all subsequent frozen embryo transfer cycles in the two groups. [file 13048_2023_1158_MOESM3_ESM.docx]

**Supplemental Table 2. Cumulative outcomes after one entire ART cycle including fresh and all subsequent frozen embryo transfer cycles in the two groups.**

|  | Case  n=443 | Control  n=7736 | P value |
| --- | --- | --- | --- |
| **Fresh cycle** |  |  |  |
| No. of retrieval cycles | 443 | 7736 |  |
| No. of fresh ET cycles | 443 | 7736 |  |
| Implantation rate (%) | 252/605(41.7%) | 5397/11124(48.5%) | **0.001** |
| Clinical pregnancy rate/ET , n (%) | 212(47.9%) | 4440(57.4%) | **<0.001** |
| Abortion rate, n (%) | 34(16.0%) | 574(12.9%) | 0.182 |
| LBR per oocytes retrieval, n (%) | 178/443(40.2%) | 3866/7736(50.0%) | **<0.001** |
| LBR per ET, n (%) | 178/443(40.2%) | 3866/7736(50.0%) | **<0.001** |
| **FET cycle** |  |  |  |
| **Cryopreservation rate, n (%)** | 240/443 | 6097/7736 |  |
| Total no. of FET with transfer | 160 | 3652 |  |
| No. of FET1 cycles | 129 | 2826 |  |
| No. of FET2 cycles | 23 | 681 |  |
| No. of FET3 cycles | 6 | 113 |  |
| No. of FET4 cycles | 2 | 26 |  |
| No. of FET5 cycles | 0 | 6 |  |
| Blastocyst transfer cycles | 125 | 3468 |  |
| Day 3 ET cycles | 35 | 184 |  |
| Clinical pregnancy rate per FET , n (%) | 73/160 (45.6%) | 2193/3652 (60.0%) | **<0.001** |
| Implantation rate | 82/195 (42.1%) | 2591/4811 (53.9%) | **0.001** |
| Abortion rate, n (%) | 21 (28.8%) | 468 (21.3%) | 0.135 |
| LBR per patient, n (%) | 52/129 (40.3%) | 1723/2826 (61.0%) | **<0.001** |
| Live birth rate per FET cycle with embryo transfer, n (%) | 52/160 (32.5%) | 1723/3652 (47.2%) | **<0.001** |

Continuous data are reported as medians (first quartile, third quartile) and analyzed by Mann-Whitney U tests.

Categorical data are reported as n (%) and analyzed by χ^2^.

LBR means live birth rate; DOR means diminished ovarian reserve; FET means frozen-thawed embryo transferred; ET means embryo transferred.
