# Supplementary material for: Diminished ovarian reserve may not be associated with a poorer fresh cycle outcome in women < 38 years
Source: J Ovarian Res. 2023 Apr 15;16:77. doi: 10.1186/s13048-023-01158-6 (PMC10105451; doi:10.1186/s13048-023-01158-6)
Supplement: Supplementary file 4 — Additional file 4: Supplemental Table 3. Singleton abnormal perinatal outcome in fresh cycles. [file 13048_2023_1158_MOESM4_ESM.docx]

**Supplemental Table 3. Singleton abnormal perinatal outcome in fresh cycles.**

|  | **Case**  **(n=141)** | **Control**  **(n=2949)** | **P value** | **P(a) value** |
| --- | --- | --- | --- | --- |
| Age | 30 (28~34) | 30 (27~32) | **0.001** | - |
| BMI | 21.8 (19.8~24.1) | 21.3 (19.6~23.4) | 0.132 | - |
| Male factor infertility | 10 (7.1) | 741 (25.1) | **<0.001** | - |
| Two embryos transferred | 36 (25.5) | 968 (32.8) | 0.071 | - |
| Blastocyst | 7 (5.0) | 347 (11.8) | **0.013** | - |
| HDP | 8 (5.7) | 74 (2.5) | **0.022** | 0.085 |
| Gestational diabetes mellitus | 14 (9.9) | 178 (6.0) | 0.061 | 0.223 |
| Abnormal placenta | 3 (2.1) | 120 (4.1) | 0.249 | 0.311 |
| Cesarean delivery | 106 (75.2) | 2038 (69.1) | 0.127 | 0.259 |
| Preterm delivery, <37 wk | 8 (5.7) | 166 (5.6) | 0.982 | 0.414 |
| Very preterm delivery, <32wk | 1 (0.7) | 16 (0.5) | 0.794 | 0.824 |
| Male gender | 72 (51.1) | 1584 (53.7) | 0.538 | 0.764 |
| Low birth weight, <2,500 g | 1 (0.7) | 36 (1.2) | 0.585 | 0.657 |
| Macrosomia, >4000 g | 11 (7.8) | 121 (4.1) | **0.034** | 0.168 |
| Fetal malformation | 1 (0.7) | 35 (1.2) | 0.606 | 0.419 |

Note: Continuous data are reported as medians (first quartile, third quartile) and analyzed by Mann-Whitney U tests.

Categorical data are reported as n (%) and analyzed by χ^2^.

P(a) values are based on binary logistic regression, adjusted for female age, BMI, primary infertility, duration of infertility in years, infertility diagnosis, COS protocols, fertilization methods, number of embryos transferred, type of embryo transferred, and from either fresh or FET cycles.

HDP= hypertensive disorders of pregnancy

^*^P<.05
